# Metformin Protects against Spinal Cord Injury and Cell Pyroptosis via AMPK/NLRP3 Inflammasome Pathway

**DOI:** 10.1155/2022/3634908

**Published:** 2022-03-27

**Authors:** Yajiang Yuan, Xiangyi Fan, Zhanpeng Guo, Zipeng Zhou, Weiran Gao

**Affiliations:** ^1^Department of Orthopedics, First Affiliated Hospital of Jinzhou Medical University, Jinzhou, China; ^2^Department of Otolaryngology-Head and Neck Surgery, First Affiliated Hospital of Jinzhou Medical University, Jinzhou, China; ^3^Department of Oncology, First Affiliated Hospital of Jinzhou Medical University, Jinzhou, China

## Abstract

Spinal cord injury (SCI) is an extreme neurological impairment with few effective drug treatments. Pyroptosis is a recently found and proven type of programmed cell death that is characterized by a reliance on inflammatory caspases and the release of a large number of proinflammatory chemicals. Pyroptosis differs from other cell death mechanisms such as apoptosis and necrosis in terms of morphological traits, incidence, and regulatory mechanism. Pyroptosis is widely involved in the occurrence and development of SCI. In-depth research on pyroptosis will help researchers better understand its involvement in the onset, progression, and prognosis of SCI, as well as provide new therapeutic prevention and treatment options. Herein, we investigated the role of AMPK-mediated activation of the NLRP3 inflammasome in the neuroprotection of MET-regulated pyroptosis. We found that MET treatment reduced NLRP3 inflammasome activation by activating phosphorylated AMPK and reduced proinflammatory cytokine (IL-1*β*, IL-6, and TNF-*α*) release. At the same time, MET improved motor function recovery in rats after SCI by reducing motor neuron loss in the anterior horn of the spinal cord. Taken together, our study confirmed that MET inhibits neuronal pyroptosis after SCI via the AMPK/NLRP3 signaling pathway, which is mostly dependent on the AMPK pathway increase, hence decreasing NLRP3 inflammasome activation.

## 1. Introduction

External direct or indirect sources are the most common causes of spinal cord injury (SCI), resulting in a permanent disability below the level of injury [1]. Primary SCI and secondary SCI are two types of acute SCI [2]. The former refers to damage to the spinal cord induced by an external force operating directly or indirectly on it, in view of the fact that secondary injury refers to spinal cord edema, intraspinal hematoma, compression fractures, and fragmentation of intervertebral disc tissue, leading to further SCI caused by spinal cord compression, resulting in a series of inflammation and oxidation. The stress response eventually leads to neuronal apoptosis [3]. Neuronal apoptosis is the source of difficult recovery of spinal cord function after SCI [4].

Metformin (MET) is a hypoglycemic drug commonly utilized in the treatment of type 2 diabetes [5], as it gets more out of hyperglycaemia without causing endogenous insulin secretion or hypoglycaemia [6]. In addition to its role in lowering blood glucose levels, MET has been shown to be a potent candidate for a variety of central nervous system (CNS) infections, including Parkinson's sickness, Alzheimer's illness, and ischemic cerebrum damage [7–9]. There is increasing evidence that the pleiotropic effects of MET may be mediated by activated AMP-activated protein kinase (AMPK), which gives signs of good effects, such as anti-inflammatory, antiapoptotic, and antioxidant properties, on diseases associated with the CNS [10–13]. A near in time learning process showed that “MET can prevent myocardial I/R damages and inhibit inflammation by activating AMPK and its regulated NOD-like receptor protein 3 (NOD-like receptor protein 3, NLRP3) inflammasome” [9].

As a newly discovered process of programmed cell death, pyroptosis has received increasingly attention to researchers, and it often takes place to various stress environments [14]. Pyroptosis can also make come about excessive cellular inflammatory damage [15, 16]. Besides necrosis, apoptosis, and autophagy, pyroptosis also plays a vital part in SCI [17]. NLRP3 inflammasome, closely related to SCI, assumes an essential administrative part in stimulating and regulating innate immune and inflammatory responses [18, 19]. A greatly sized number of studies have shown that NLRP3 inflammasome plays a crucial regulatory role in the occurrence and progression of SCI [2, 19]. Pyroptosis is a favorable to provocative related customized cell demise pathway that significantly affects neuroinflammation, which is a key element driving optional injury after SCI and activated by inflammasome. Pyroptosis can lead to a variety of neurological issues, including neurodegenerative infections, ischemic cerebrum injury, and psychiatric disorders. In addition, pyroptosis can aggravate the death of neurons and glial cells after SCI. Therefore, it is of extraordinary importance to track down a protected and viable medication to target pyroptosis after SCI. Currently clinically used drugs such as methyl prednisolone used to be the standard treatment for acute SCI due to its anti-inflammatory properties, but these drugs have adverse side effects, such as sepsis, gastrointestinal bleeding, and thromboembolism. Therefore, it is of great significance to find a safe and effective drug to target pyroptosis after SCI. Although previous studies have confirmed that metformin's beneficial effect on SCI improves functional recovery from autophagy flux stimulation [20], the role of autophagy remains unclear, and it has both beneficial and harmful aspects. Meanwhile, studies have confirmed that “metformin protects against myocardial ischemia-reperfusion injury and pyroptosis through the AMPK/NLRP3 inflammasome pathway” [9]. This gives us a revelation that metformin is effective against pyroptosis. However, there are no studies on the impact of MET on the activation of NLRP3 inflammasome in SCI.

The capacity of MET to protect neurons from acute SCI damage was studied in this study, as well as whether the protective mechanism of MET involves the modulation of the AMPK/NLRP3 pathway.

## 2. Methods

### 2.1. Cell Culture

Primary neurons were removed from the spinal cord of newborn (<48 h) rat pups, the neonatal mouse pups were euthanized by rapid neck-breaking, and the tissues were minced and gathered and afterward processed with EDTA containing 0.25% trypsin for 15 min at 37°C. After the absorption was ended, the cells were cultivated onto poly-DL-lysine-coated glass coverslips (30-70 kDa, Sigma-Aldrich) in 24-well plates and refined in Dulbecco's modified Eagle's medium (DMEM) containing 10% foetal bovine serum (FBS) and 25 mg/ml penicillin/streptomycin (Gibco Life Technologies, Carlsbad, CA, USA). RAW 264.7 cells (procured from the American Type Culture Collection (ATCC)) were refined in DMEM medium containing 10% FBS. All cells were grown at 37°C in 5% CO_2_. The cells were haphazardly partitioned into 4 groups: (1) control group: no additional treatment was added to the cell culture mediums, (2) lipopolysaccharide (LPS) group: 100 ng/mL LPS was added to the cell culture mediums for stimulation for 24 h, (3) MET group: 50 *μ*M of MET was added to the culture medium after the treatment with LPS, and (4) MET+compound C (CC) group: 50 *μ*M of MET and 50 *μ*M of CC were added after the treatment with LPS. The selection of drug dosage was based on previous studies [20].

### 2.2. Cell Viability

The decrease of 3-(4,5-dimethylthiazol-2-yl)-2,5-diphenyltetrazolium bromide (MTT) to a purple formazan item was measured to decide the impact of MET on cell survival. In short, after LPS induction, the cells were treated with MET (0, 1, 10, 50, 100, 200, and 500 *μ*M). Then, at that point, after 24 h hatching, MTT (20 *μ*L, Sigma-Aldrich) was added to each well, and the plates were kept under the right conditions for growth for 4 h at 37°C. Next, 150 *μ*L dimethyl sulfoxide (DMSO, Sigma-Aldrich) was added for 10 min at 37°C to dissolve the crystals. Finally, a microplate peruser was utilized to gauge and record the absorbance at 490 nm.

### 2.3. Western Blot

Western blot methods were performed according to previously studied [21]. The accompanying antibodies were utilized for recognition in this experiment: antiphosphorylated AMPK (p-AMPK, 1 : 1000, Cell Signaling Technology, USA), anti-AMPK (1 : 1000, Cell Signaling Technology), anti-p-ACC (1 : 1000, Cell Signaling Technology), anti-ACC (1 : 1000, Cell Signaling Technology), anti-NLRP3 (1 : 1000, Cell Signaling Technology), anti-ASC (1 : 1000, Abcam, UK), anti-Caspase 1 (1 : 1000, Abcam), anti-IL-1*β* (1 : 1000, Abcam), anti-GAPDH (1 : 1000, Proteintech, USA), HRP Affinipure Goat Anti-Mouse IgG (1 : 1000, Proteintech), and HRP Affinipure Goat Anti-Rabbit IgG (1 : 1000, Proteintech). The outcomes were investigated with an UVP Electrophoresis Gel Imaging System (US) and ImageJ programming.

### 2.4. Confocal Fluorescence Imaging

Primary neurons and RAW 264.7 cells filled in a confocal petri dish were washed with super cold PBS (4°C, 0.1%, 3 × 5 min) and fixed with super cold 4% paraformaldehyde for 30 min. After the cells were washed with PBS for multiple times, they were impeded with typical goat serum containing 0.1% Triton X-100 for 2 h at 4°C. The cells were then incubated overnight at 4°C with the appropriate anti-NLRP3 (1 : 200, Cell Signaling Technology), anti-p-AMPK (1 : 200, Cell Signaling Technology), anti-NeuN (1 : 200, Abcam), or anti-*β*-Tubulin (1 : 1000, Proteintech) followed by washes with PBS and incubation with an Alexa Fluor®488-conjugated goat anti-mouse/rabbit IgG antibody for 2 h at room temperature. 4,6-Diamidino-2-phenylindole (DAPI, 1 : 1,000, Abcam) was used to stain nuclei. A high-resolution confocal microscope was used to observe the cells (Leica, Heidelberger, Germany) and examined by the ImageJ software.

### 2.5. Inflammatory Cytokine Measurement

The enzyme-linked immunosorbent assay (ELISA) kit (Solarbio, China) was utilized to recognize the inflammatory cytokines interleukin-1*β* (IL-1*β*), interleukin-6 (IL-6), and tumor necrosis factor-*α* (TNF-*α*) in cell supernatant or serum concentration. The operation method of the ELISA kit was in accordance with the instruction of the instruction manual.

### 2.6. Animals and Drug Treatment

Sprague-Dawley (SD) female rats (8 weeks, 180-220 g) were bought from Liaoning Changsheng Biotechnology Co., Ltd. (Benxi, China). All experimental procedures complied with the Care and Use of Laboratory Animals guidelines published by the US National Institutes of Health and were approved by the Laboratory Animal Ethics Committee of Jinzhou Medical University. Animals were reared at a temperature 23 ± 0.5°C with day and night light to simulate the natural environment. The test creatures were partitioned into 3 gatherings similarly: the Sham group, which underwent laminectomy only, the Vehicle group, which went through a medical procedure for SCI and got Vehicle (0.9% saline, i.p.) right away and 24 hours after surgery, and the MET group, which went through a medical procedure for SCI and got MET (50 mg/kg, i.p.) right away and 24 hours after surgery. The portions of MET administered depended on past studies [20] and guided by the producers' instructions.

### 2.7. Spinal Cord Injury Model

Anaesthetized (3% sodium pentobarbital, 40 mg/kg, i.p., Solarbio) rats were put in a prone position on a cork platform. There was no rat displayed indications of peritonitis, torment, or inconvenience following i.p. administration of sodium pentobarbital. The surgery was performed under sterile conditions, and the T9/10 lamina was excised after incision of the skin. The SCI model was built using Allen's technique [22]. The impactor (diameter 2 mm, 10 g, and 25 mm height) was dropped rapidly to impact the spinal cord to cause injury. After successful modeling, the wound was sutured layer by layer. The rats were assisted in massaging their bladders three times a day until they were able to void spontaneously again. When taking the materials, excessive pentobarbital (150 mg/kg) were injected peritoneal to euthanize the rats.

### 2.8. Locomotion Recovery Assessment

In an open field, the Basso, Beattie, and Bresnahan (BBB) scores and the inclined plane test were administered on days 0, 1, 3, 7, 14, 21, and 28 after SCI to assess motor recovery in all animals. All tests were performed by specialists double-blinded to the treatments.

### 2.9. Hematoxylin-Eosin (HE) Staining

After 28 days, the rats were perfused with 0.9% saline and 4% paraformaldehyde, and the spinal cord tissue was extracted. The spinal line fragments, including the injury site, were cut utilizing a cryostat (Leica CM3050S, Heidelberg, Germany) (10 *μ*m transverse cryosections). After HE staining, histological alterations at the injury site were evaluated, and the number of anterior horn motor neurons was counted.

### 2.10. Immunohistochemical Analysis

Immunohistochemistry analysis was used to detect the subcellular localization of NLRP3. Spinal line paraffin segment was deparaffinized and rehydrated. In the wake of washing with Tris-buffered saline (TBS), endogenous peroxidase action and vague antigens were hindered with 5% typical bovine serum. The segment was then treated overnight at 4°C with anti-NLRP3 (1 : 200, Cell Signaling Technology). The primary antibody was not used in the negative controls. After then, a biotinylated anti-rabbit IgG antibody was used to bind the section, which was subsequently followed by HRP-conjugated streptavidin. A biological imaging microscope was used to capture the images. The result of immunohistochemical analysis is the optical density value of the picture (optical density), namely, the OD value. The deeper the immunohistochemical DAB staining, the greater the OD value and the more protein expression. The Image J software was used to determine the OD value obtained by DAB staining, and the outcomes were analyzed utilizing the SPSS 19.0 software.

### 2.11. Statistical Analysis

The mean ± standard error of the mean (SEM) was used to communicate the data, which was broken down using SPSS 19.0 programming. To make a correlation of the differences across different gatherings, one-way analysis of variance (ANOVA) or two-way inquiry of fluctuation was used, followed by Bonferroni's post hoc test. *P* values < 0.05 were considered as genuinely critical.

## 3. Results

### 3.1. Selection of Metformin Concentrations for the LPS-Treated Neuron Model

**Figure 1:**
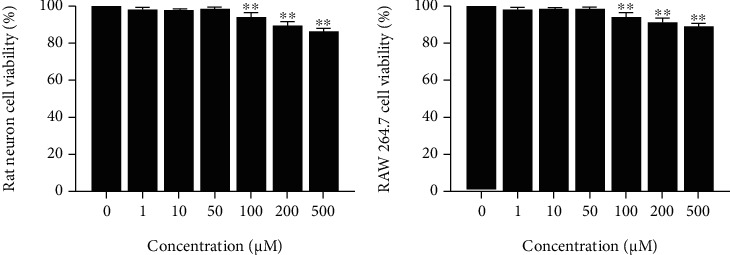
Representative MTT assay shows the viability of (a) primary spinal cord neurons and (b) RAW 264.7 cells incubated with metformin (0, 1, 10, 50, 100, 200, and 500 *μ*M) for 24 h. ^∗∗^*P* < 0.01 versus the control group, *n* = 5.

The MTT assay was performed to determine cell viability after cells were treated with MET (0, 1, 10, 50, 100, 200, and 500 M) for 24 hours. Rat neuronal cell (Figure 1(a)) and RAW 264.7 cell (Figure 1(b)) viability showed a decrease when the concentration was higher than 100 *μ*M MET treatment (Figure 1, ^∗∗^*P* < 0.01, *n* = 5); therefore, a dose of 50 *μ*M was selected for all subsequent experiments.

### 3.2. Metformin Activates the AMPK Signaling Pathway and Suppresses Pyroptosis in Neuronal Cells

**Figure 2:**
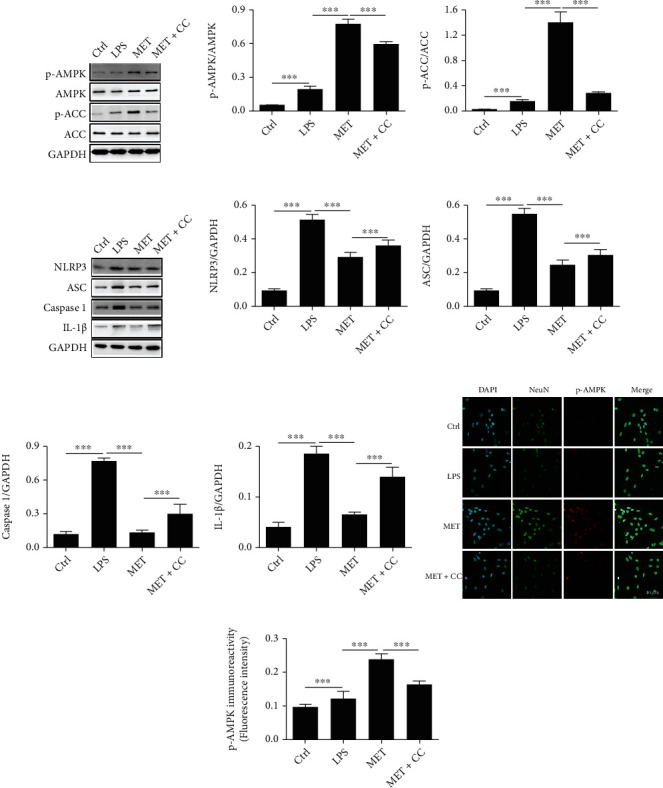
Metformin activates the AMPK/NLRP3 signaling pathway in spinal cord neurons. (a–c) Representative Western blots and quantification data of the levels of p-AMPK and p-ACC in the spinal cord neurons from each group. All data represented the mean ± SD, ^∗∗∗^*P* < 0.001, *n* = 3. (d–h) Representative Western blots and quantification data of the expression of NLRP3, ASC, Caspase 1, and IL-1*β* in the spinal cord neurons from each group. All data represent the mean ± SD, ^∗∗∗^*P* < 0.001, *n* = 3. (i, j) Immunofluorescence examination was utilized to recognize the staining power of antibodies focusing on NeuN and p-AMPK. Scale bars represent 40 *μ*m. ^∗∗∗^*P* < 0.001, *n* = 3.

Primary neurons were treated with LPS to establish a model of injury. To demonstrate the effect of MET on the AMPK/NLRP3 signaling pathway *in vitro*, MET and CC were added in the cells. Then, the levels of p-AMPK, AMPK, p-ACC, ACC, NLRP3, ASC, Caspase 1, and IL-1*β* were performed by Western blot analysis. As shown in Figure 2(a), the AMPK/ACC axis was activated by MET, which elevated the levels of p-AMPK. At the same time, this change was eliminated by CC (Figures 2(a)–2(c), ^∗∗∗^*P* < 0.001, *n* = 3).

The expression of NLRP3, ASC, Caspase 1, and IL-1 was considerably downregulated after LPS-induced cells were treated with MET (Figures 2(d)–2(h), ^∗∗∗^*P* < 0.01, *n* = 3). In addition, immunofluorescence showed that the levels of p-AMPK were significantly inhibited by MET in LPS-treated primary spinal cord neurons, and CC reversed this phenomenon (Figures 2(i) and 2(j), ^∗∗∗^*P* < 0.01, *n* = 3). These findings imply that MET activates the AMPK/NLRP3 signaling pathway and suppresses pyroptosis.

### 3.3. Metformin Inhibits Inflammatory Expression *In Vitro*

**Figure 3:**
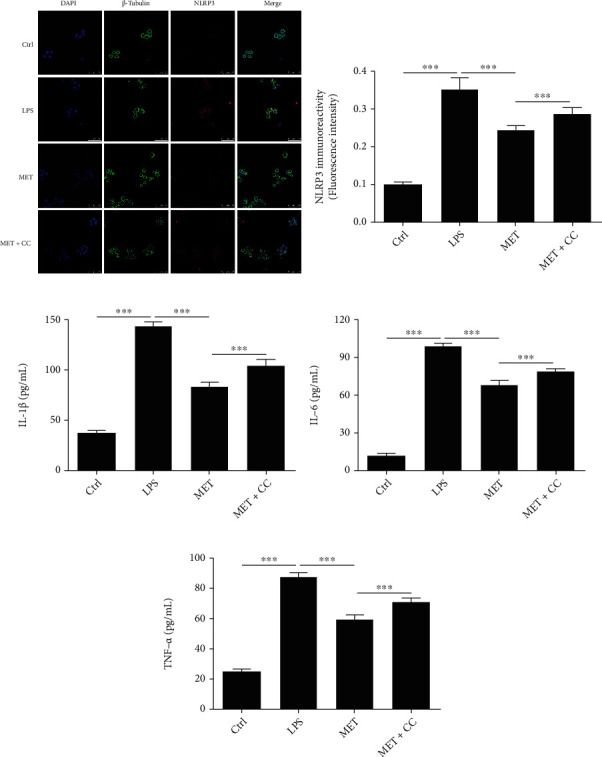
Metformin inhibits NLRP3 activation as well as proinflammatory factor expression in RAW 264.7 cells. (a, b) Immunofluorescence staining intensity of antibodies targeting NLRP3 in RAW 264.7 cells was determined using immunofluorescence analysis. Scale bars represent 50 *μ*m. ^∗∗∗^*P* < 0.001, *n* = 3. (c–e) ELISA was used to detect the expression of IL-1*β*, IL-6, and TNF-*α* in the supernatant of RAW 264.7 cells from each group. All data represent the mean ± SD, ^∗∗∗^*P* < 0.001, *n* = 3.

To explore the ability of MET to inhibit inflammation, RAW 264.7 cells treated with LPS were used as an inflammation model *in vitro*. The expression of the NLRP3 inflammasome was reduced by MET therapy, as illustrated in Figures 3(a) and 3(b). At the same time, the addition of CC increased the expression of NLRP3. This is consistent with the performance of primary neurons. To further explore the anti-inflammatory ability of MET, ELISA evaluated the levels of IL-1*β*, IL-6, and TNF-*α* in the supernatant of RAW 264.7 cells. IL-1*β*, IL-6, and TNF-*α* levels increased in all groups except the control group (Figures 3(c)-3(e), ^∗∗∗^*P* < 0.01, *n* = 3). The MET group's inflammation level was lower than the LPS group's, whereas the MET+CC group's inflammation level was higher than the MET group's. We concluded that MET significantly prevented LPS-induced upregulation of inflammatory factor levels. This could be linked to MET's regulation of NLRP3 expression.

### 3.4. Metformin Improves Functional Recovery after SCI

**Figure 4:**
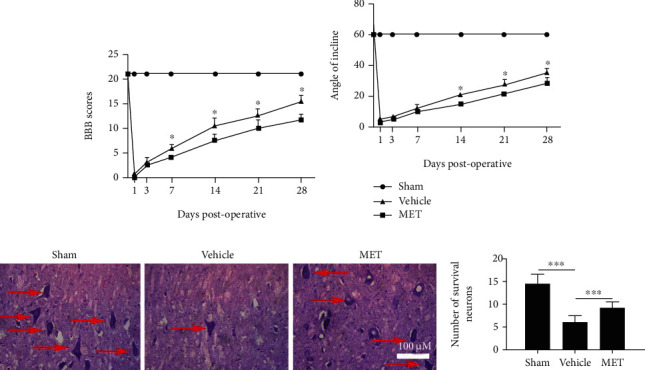
Metformin works on practical recuperation and diminishes the deficiency of neurons following SCI. (a) Scores of Basso, Beattie, and Bresnahan (BBB), ^∗^*P* < 0.05, *n* = 3. (b) The inclined plane test, ^∗^*P* < 0.05, *n* = 3. (c) HE staining at 28 days. Scale bars represent 100 *μ*m. (d) Counts of the number of front corner motor neurons. All data represent the mean ± SD, ^∗∗∗^*P* < 0.001, *n* = 3.

The BBB scores served as a proxy for the beneficial effect of MET on locomotor recovery following SCI. The BBB score of the Sham group was normal, and there was no measurable difference between the MET group and the Vehicle group at 1 and 3 days after injury. Regardless, the scores in the MET group were higher 7 days after SCI (Figure 4(a), ^∗^*P* < 0.05, *n* = 3). The results of the inclined plane test were similar to the BBB scores (Figure 4(b), ^∗^*P* < 0.05, *n* = 3). These findings indicated that MET can improve motor function following SCI.

### 3.5. Metformin Reduces the Loss of Neurons following SCI

On day 28 after SCI, HE staining revealed changes in the neurons of the ventral horn of the spinal cord. The number of ventral horn motor neurons was considerably larger in the MET group than in the Vehicle group (Figures 4(c) and 4(d), ^∗∗∗^*P* < 0.001, *n* = 3). These results indicated that MET was able to reduce motor neuron death after SCI.

### 3.6. Metformin Activates the AMPK Signaling Pathway and Reduces Pyroptosis after SCI

**Figure 5:**
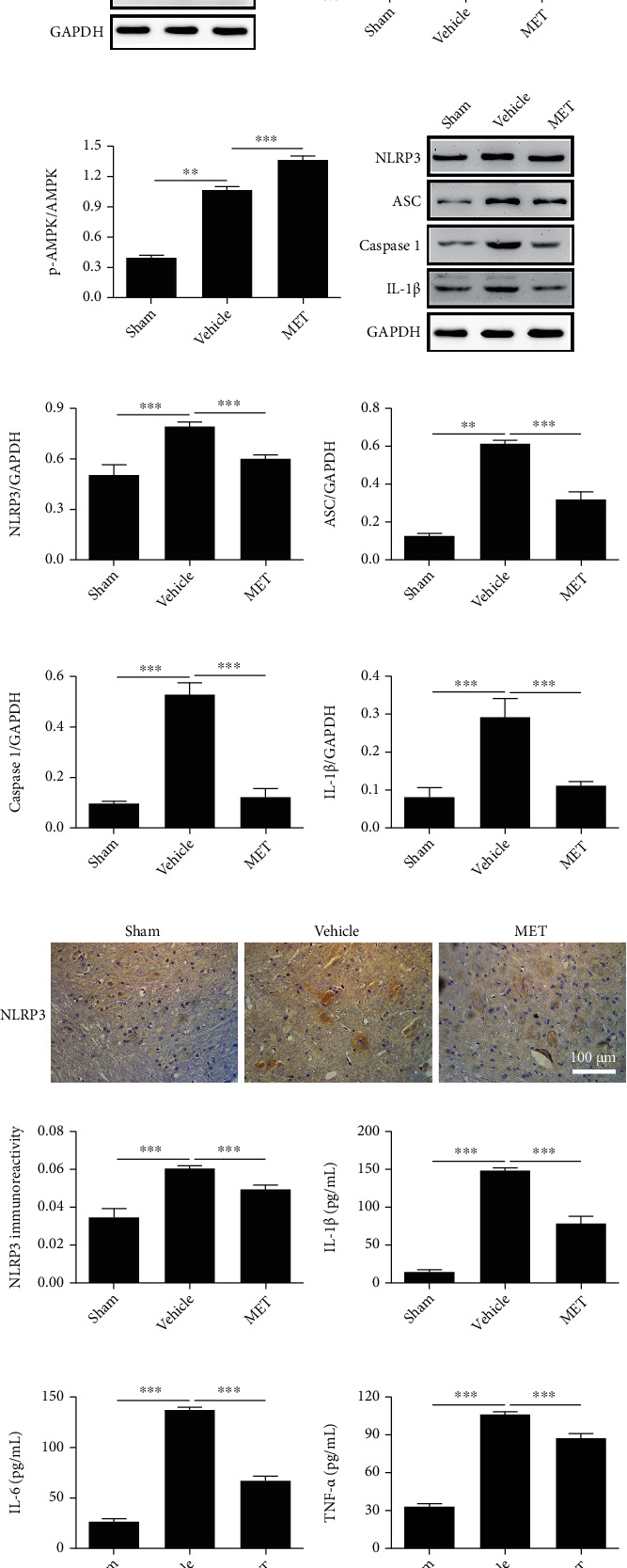
Metformin activates the AMPK/NLRP3 signaling pathway and inhibits inflammation after SCI. (a–c) Representative Western blots and quantification data of the levels of p-AMPK and p-ACC after SCI from each group. All data represent the mean ± SD, ^∗∗∗^*P* < 0.001, *n* = 3. (d–h) Representative Western blots and quantification data of the expression of NLRP3, ASC, Caspase 1, and IL-1*β* after SCI from each group. All data represent the mean ± SD, ^∗∗∗^*P* < 0.001, *n* = 3. (i, j) Immunohistochemical detection of the levels of NLRP3 protein in each group of rats at 7 days after SCI. Scale bars represent 100 *μ*m. ^∗∗∗^*P* < 0.001, *n* = 3. (k–m) ELISA was used to detect the expression of IL-1*β*, IL-6, and TNF-*α* in the spinal cord tissue homogenate from each group. All data represent the mean ± SD, ^∗∗∗^*P* < 0.001, *n* = 3.

To determine whether the AMPK pathway is taken part with the effects of MET treatment after SCI, Western blot was used to look for the levels of p-AMPK, AMPK, p-ACC, and ACC. The MET treatment group had significantly increasing levels of p-AMPK and p-ACC than the Vehicle group (Figures 5(a)–5(c), ^∗∗∗^*P* < 0.001, *n* = 3). Next, we examined whether MET could reduce pyroptosis *in vivo* by activating AMPK phosphorylation. As shown in Figures 5(d)–5(h), SCI-induced pyroptosis was manifested by increased expression levels of NLRP3, ASC, Caspase 1, and IL-1*β* and a significant decrease in MET group (^∗∗∗^*P* < 0.001). MET inhibited SCI-induced activation of NLRP3 complex. Furthermore, immunohistochemistry was utilized to distinguish the levels of NLRP3 protein after SCI, which were importantly dropped in the MET treatment group compared with the levels in the other groups (Figures 5(i) and 5(j), ^∗∗∗^*P* < 0.001, *n* = 3). These outcomes showed that MET could activate the AMPK/NLRP3 signaling pathway and reduce pyroptosis after SCI.

### 3.7. Metformin Decreases the Release of Proinflammatory Factors

The effect of MET on the release of inflammation-related cytokines after SCI was investigated in this experiment. In the spinal cord tissue homogenates, ELISA measured the amounts of IL-1*β*, IL-6, and TNF-*α*. Except for the Sham group, the levels of IL-1*β*, IL-6, and TNF-*α* rose in all groups (Figures 5(k)–5(m), ^∗∗∗^*P* < 0.001, *n* = 3). In comparison to the Vehicle group, the MET group had lower levels of inflammatory factors (Figures 5(k)–5(m), ^∗∗∗^*P* < 0.001, *n* = 3). These findings indicated that MET inhibited the release of proinflammatory factors after SCI.

## 4. Discussion

As far as we have knowledge of, this is the first learning process to investigate the regulation of AMPK/NLRP3 inflammasome pathway by MET after SCI. We found that MET has a neuroprotective effect *in vivo* and *in vitro* by inhibiting nerve cell pyroptosis and inflammation. MET is a biguanide hypoglycaemic agent normally used to treat type 2 diabetes [6]. A large number of studies have made clear that MET puts power into neuroprotective effects on some CNS diseases and neurodegenerative diseases [7, 8, 11]. MET activates AMPK through STK11 (also experienced as LKB1), which has to do with multiple signaling pathways and affects cell growth, development, apoptosis, and other biological functions [13, 23, 24]. AMPK is the main regulator of cellular energy producing and the interaction between metabolism and tumors [25, 26].

“Pyroptosis, also experienced as cell inflammatory necrosis, is a new way of programmed cell death discovered and confirmed in recent years” [14, 27]. It is clearly manifested in the swelling and rupture of cells, leading to the outflow of cellular contents and the release of a large number of inflammatory factors [28]. Pyroptosis is a significant normal resistant reaction of the body, which has a significant impact in the battle against disease. “Pyroptosis is a gasdermin-mediated programmed cell necrosis” [27]. The morphological qualities, event, and guideline instrument of pyroptosis are not the same as other cell death methods like apoptosis and necrosis [28]. Studies have given view that pyroptosis is significant in the occurrence event and improvement of growths, irresistible illnesses, metabolic infections, and sensory system-related sicknesses [29]. Further investigation of pyroptosis will assist with getting past information on its event and advancement in related sicknesses and its part in visualization and give groundbreaking thoughts for clinical counteraction and therapy. The course of pyroptosis is Caspase 1 dependent [30]. Under the feeling of outside conditions, the precursor of Caspase 1 can form a polymer complexes with the pattern recognition receptors NLRP1 and NLRP3 through the adaptor protein ASC, namely, the inflammasome, also experienced as caspase-dependent inflammasome [31, 32]. At the point when Caspase 1 is initiated, cells will deliver the inflammatory factors IL-1*β* and IL-18, which will stand out from more provocative cells and aggravate the inflammatory response. The inflammasome is a multiprotein complex that plays a direct role in the inflammatory process by causing Caspase 1, IL-1, and IL-18 to become overactive. Inflammation can worsen tissue necrosis and apoptosis following SCI, according to studies, and the NLRP3 inflammasome plays a key role in this [18, 33, 34]. NLRP3 is the most studied inflammatory complex protein in vivo, as it is a member of the inflammasome nod-like receptor (NLR) family [32]. NLRP3 is a significant effector in the inflammatory response and has a role in the development of SCI [18]. There appears to be a link between AMPK, mTOR, and NLRP3 inflammasome signals, according to research. Actuation of AMPK diminishes the action of mTOR and NLRP3 [35, 36].

Ischemia and hypoxia, immunological inflammation, free radical damage, and lipid peroxidation are all pathogenic events in secondary SCI [3]. Prevention and intervention of secondary injuries is taken into account as a hoping treatment for SCI. Among the pathophysiological occasions of secondary injury mentioned, cell death and inflammation are considered the two key goals of SCI treatment [37]. A growing number of researches have revealed that necroptosis and pyroptosis can prompt an assortment of neurological sicknesses, including neurodegenerative illnesses, ischemic mind injury, and mental infections [38]. In addition, necroptosis and pyroptosis can exacerbate the death of neurons and glial cells after SCI [18]. Along these lines, improving AMPK action and its inhibitory impact on NLRP3 motioning to target irritation and pyrolysis is relied upon to turn into an intriguing treatment for SCI.

In this study, primary neurons and RAW 264.7 cells were used to simulate the SCI model *in vitro*. We found that MET treatment activated AMPK and its downstream protein ACC and restrained NLRP3 inflammasome enactment in primary neurons. The AMPK inhibitor CC reversed the suppression of pyroptosis-related proteins caused by MET therapy. At the same time, macrophages/microglias are effector cells of the immune response in CNS [39], so RAW 264.7 cells were used to simulate inflammatory damage. The expression of proinflammatory factors such as IL-1*β*, IL-6, and TNF-*α* was shown to be considerably suppressed by MET. These findings suggest that MET has a powerful anti-inflammatory impact, which is associated with actuation of AMPK and restraint of NLRPR initiation.

To further verify the mechanism of action of MET *in vivo*. We established SCI model of rats. We found that MET treatment reduced neuronal loss and improved motor function recovery after SCI. At the same time, MET treatment activated the spinal cord tissue AMPK signaling pathway and inhibited NLRP3 inflammasome activation. ELISA results also confirmed that MET inhibited the expression of inflammatory factors. These results are consistent with those of *in vitro* experiments.

The molecular mechanism of MET treatment of SCI is complex, involving multiple molecular pathways. Our study showed that MET inhibited pyroptosis and inhibited inflammatory responses after SCI by activating AMPK and its regulated NLRP3 inflammasome. Therefore, the regulation of NLRP3 by AMPK may give another helpful idea and strategy for the treatment of SCI.

## 5. Conclusions

Ultimately, this research is the first to establish that metformin hinders neuronal pyroptosis and works on utilitarian recuperation after SCI through the AMPK/NLRP3 signaling pathway. Our outcomes suggest that metformin might serve as a therapeutic intervention to reduce inflammation and the effects of acute SCI.

## Data Availability

The datasets used and/or analyzed during the current study are available from the corresponding author on reasonable request.
