# Correction to “Metformin Protects against Spinal Cord Injury and Cell Pyroptosis via AMPK/NLRP3 Inflammasome Pathway”

**DOI:** 10.1155/ancp/9838646

**Published:** 2026-05-04

Y. Yuan, X. Fan, Z. Guo, Z. Zhou, and W. Gao, “Metformin Protects against Spinal Cord Injury and Cell Pyroptosis via AMPK/NLRP3 Inflammasome Pathway,” *Analytical Cellular Pathology* 2022, no. 1 (2022): 3634908, https://doi.org/10.1155/2022/3634908.

In the above article, the middle panel depicting “Vehicle” in Figure 4c is incorrect due to an error made during figure preparation. The correct Figure 4 is as follows:

**Figure 4:**
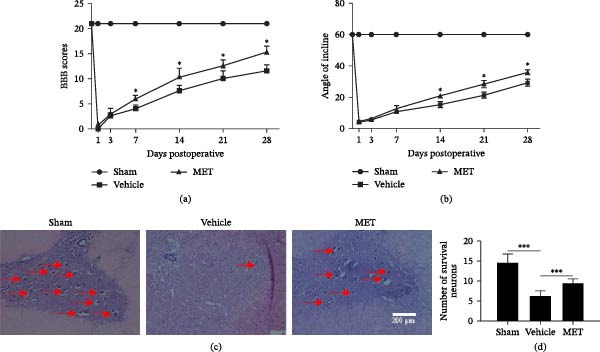
Metformin works on practical recuperation and diminishes the deficiency of neurons following SCI. (a) Scores of Basso, Beattie, and Bresnahan (BBB),  ^∗^ *p* < 0.05, *n* = 3. (b) Inclined plane test,  ^∗^ *p* < 0.05, *n* = 3. (c) HE staining at 28 days. Scale bars represent 200 μm. (d) Counts of the number of front corner motor neurons. All data represent the mean ± SD,  ^∗∗∗^ *p* < 0.001, *n* = 3.

We apologize for this error.

